# Hybrid Surgery to Manage Aortic Arch Pathology

**DOI:** 10.3390/medicina57090909

**Published:** 2021-08-30

**Authors:** Guido Gelpi, Claudia Romagnoni, Francesco Epifani, Monica Contino, Carlo Antona

**Affiliations:** 1 ASST Fatebenefratelli Sacco—Cardiovascular Surgery Department, 20100 Milano, Italy; claudia.romagnoni@asst-fbf-sacco.it (C.R.); francesco.epifani@asst-fbf-sacco.it (F.E.); monica.contino@asst-fbf-sacco.it (M.C.); carlo.antona@asst-fbf-sacco.it (C.A.); 2 Dipartimento di Scienze Biomediche e Cliniche L. Sacco, Università degli Studi di Milano, 20100 Milano, Italy

**Keywords:** aortic arch aneurysm, TEVAR, debranching, hybrid surgery

## Abstract

*Background and Objectives*: Aortic arch disease is still a high-risk surgical challenge despite major advances both in surgical and anesthesiological management. A combined surgical and endovascular approach has been proposed for aortic arch disease treatment to avoid hypothermia and circulatory arrest in high-risk patients. *Materials and Methods*: Between June 2004 and June 2021, 112 patients were referred to our department for aortic arch surgery; 38 (33.9%) patients underwent supra-aortic debranching and endovascular treatment. Of these, 21 (55%) patients underwent type I aortic arch hybrid debranching procedure and in 17 (45%) patients a type II aortic arch hybrid debranching procedure was performed. None of the patients were emergent. *Results*: No intra-operative deaths were recorded. In the type I aortic arch hybrid debranching patients’ group, one patient died at home waiting the endovascular step, one developed ascending aortic dissection and another one developed a pseudoaneurysm at the site of the debranching at follow-up. In the type II aortic arch hybrid debranching patients’ group, left carotid artery branch closure was detected at follow-up in one patient. Thirty day/in-hospital rates of adverse neurological events for both the surgical and endovascular procedures were 3% for minor stroke, with no permanent neurological deficit and 0% for permanent paraplegia/paraparesis. In 100% of the cases, the endovascular step succeeded and the type Ia endoleak rate was 0%. *Conclusions*: Hybrid arch surgery is a valuable option for aortic arch aneurysm treatment in patients with high surgical risk. The choice of aortic arch debranching between type I or type II is crucial and depends on anatomic and clinical patient characteristics. Further larger scale studies are needed to better define the advantages of these techniques.

## 1. Introduction

Aortic arch diseases are a hard entity to treat particularly in acute settings. In the last decade, the introduction of technical improvements and new surgical procedures, such as frozen elephant trunk, have improved the outcomes of open repair of the aortic arch and reduced mortality [1]. However, particularly in the elderly population, mortality rates and major neurologic injuries of aortic arch surgery are still not negligible [2].

Following the advent of transcatheter endovascular aortic repair (TEVAR) a combined vascular and endovascular approach has been proposed for aortic arch disease treatment to avoid hypothermia and circulatory arrest.

The hybrid arch concept is essentially a supra-aortic debranching to create a proximal landing zone in Ishimaru [3] arch zone 0–1 for TEVAR. In the current report, we describe our experience with hybrid treatment of aortic arch pathology focusing on different hybrid techniques and on the advantages and disadvantages of these procedures.

Based on Bavaria and co-authors’ [4] classification of aortic arch hybrid repair, the arch anatomy and the TEVAR landing zones dictate the type of the procedure. If there are adequate native proximal zone 0 and distal zone 3 and zone 4 landing zones, as in the isolated aortic arch aneurysm, a type I arch hybrid procedure is performed. The innominate artery, left common carotid artery and left subclavian artery are debranched on the native ascending aorta to enable zone 0 stent grafting, followed by concomitant antegrade or delayed retrograde TEVAR. We consider native ascending aorta as an adequate proximal landing zone if the maximum diameter is less than 3.7 cm, the length from the sinotubular junction to the beginning of the arch curvature is longer than 6 cm, on the outer side of the ascending aorta and no plaques or blister are detected on the CT scan. For arch aneurysm without a good ascending aorta that effects a safe proximal zone 0 landing zone, but an adequate zone 3/zone 4 distal landing zone, type II arch hybrid repair is performed. Therefore, the open procedure involves not only epiaortic vessel debranching, but the creation of a proximal zone 0 landing zone by replacing the ascending aorta with a Dacron graft prosthesis.

## 2. Materials and Methods

Between June 2004 and June 2021, 112 patients came to our attention for aortic arch surgery. Conventional surgery was performed in 74 (66.1%) cases, while the hybrid approach was preferred in the remaining 38 (33.9%) because of cardiovascular, neurologic or pulmonary comorbidities causing high surgical risk. Patient characteristics are shown in Table 1. Aortic pathologies were distributed as follows: aortic arch aneurysm (78%), false lumen dilation of a previous treated type A dissection (14%), penetrating aortic ulcer (8%). A total of 21 (55%) patients underwent a type I aortic arch hybrid debranching procedure and in 17 (45%) patients a type II aortic arch hybrid debranching procedure was performed. Concomitant procedures were coronary artery bypass in one patient and aortic valve repair in two cases. Contrast CT scan was always performed preoperatively to better plan the surgical and endovascular steps (disease extension, endovascular stent measures, supra-aortic vessel anatomy and vascular access feasibility). Patients also underwent coronary catheterization and echocardiogram. The study received Ethics Committee approval.

### 2.1. Surgical Technique

Standard general anesthesia is always associated with non-invasive monitoring of cerebral oxygen saturation by near-infrared spectroscopy for continuous cerebral monitoring during the procedure. Usually a median sternotomy is performed, but also a mini-sternotomy at the third intercostal space as an inverted T shape allows good exposure and mobilization of the ascending aorta and supra-aortic vessels.

### 2.2. Type I Debranching

**Figure 1:**
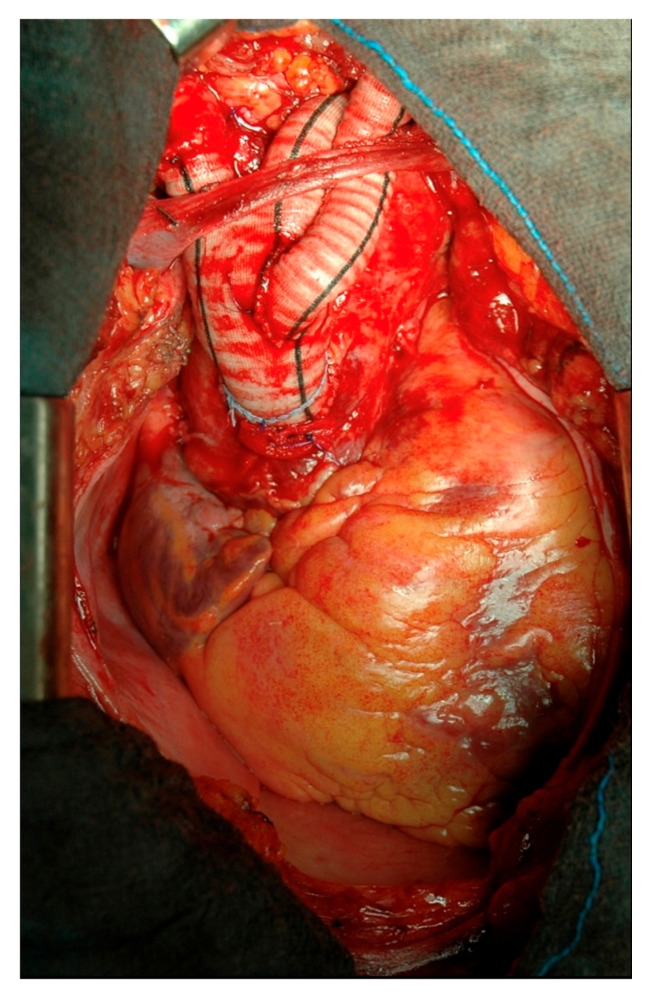
Type I aortic arch debranching.

For type I arch debranching, heparinization is started to reach a coagulation activated time longer than 250 s. After reaching a mean blood pressure of 80 mmHg by pharmacological therapy or by ventricular pacing, the ascending aorta is tangentially clamped with a side-biting clamp and the proximal part of a bi- or trifurcated Dacron vascular prosthesis (Uni-Graft K-DV; Aesculap, Tuttlingen, Germany) is sutured end-to-side to the aorta. Preoperative CT scan, transesophageal echocardiography and digital inspection are the tools to determine the position on the ascending aorta for proximal anastomosis, in order to avoid atherosclerotic plaques and to obtain an adequate landing zone for the TEVAR. Once the proximal anastomosis is completed, the first limb of the branched graft is usually anastomosed directly end-to-end to the proximal left subclavian artery when it is easily reached directly. Keeping a mean arterial pressure of over 100 mmHg for a better cerebrovascular perfusion, the second limb is then anastomosed end-to-end to the left common carotid artery and finally, the third limb of the prosthesis is anastomosed to the innominate artery, in the same fashion, (Figure 1).

**Figure 2:**
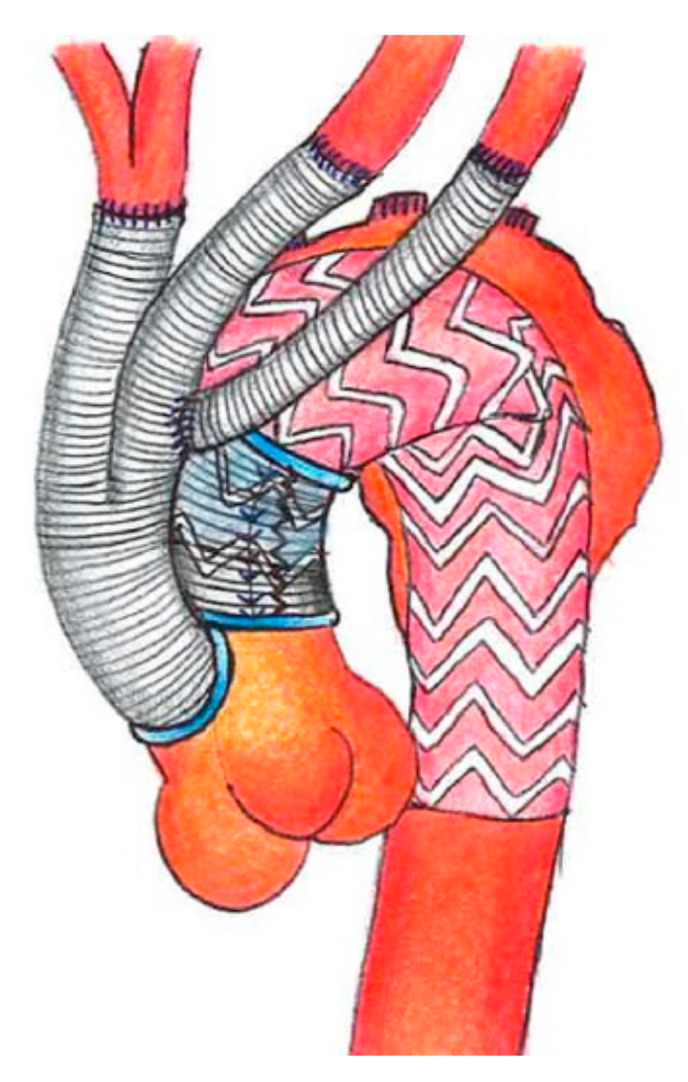
Scheme of type I debranching and aortic reinforcement.

The procedure is mostly completed by reinforcing the proximal aorta, immediately after the origin of the main trunk of the new supra-aortic vessels. The reinforcement is generally 4 cm long and the very proximal part of the bi-trifurcated graft, that is not used for the debranching, is opened longitudinally and wrapped around the aorta [5,6]. This location is chosen for where the proximal part of the future TEVAR is intended to be deployed (Figure 2).

### 2.3. Type II Debranching

**Figure 3:**
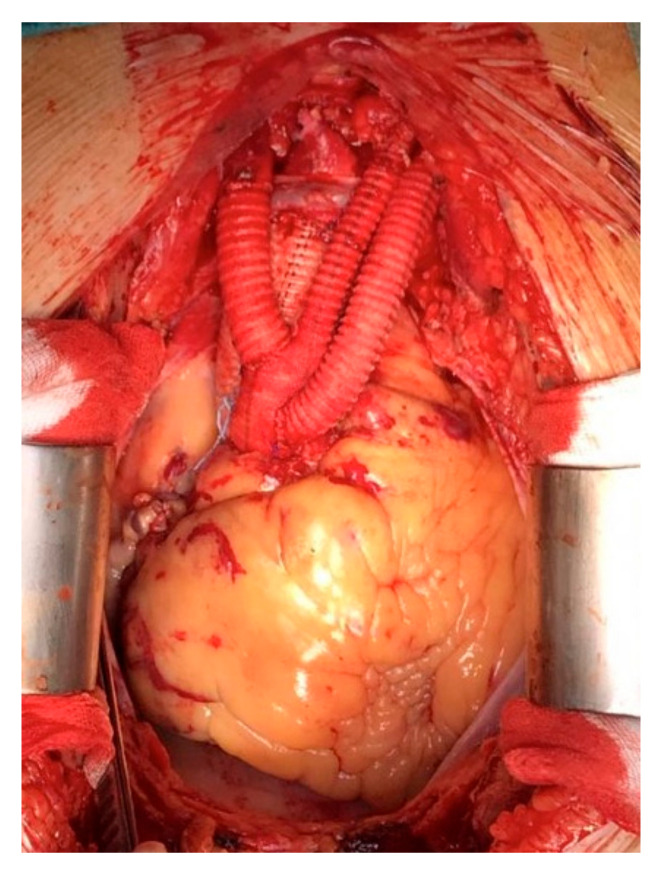
Type II aortic arch debranching.

For type II arch debranching with ascending aorta replacement, patients are placed on cardiopulmonary bypass. The arterial cannulation is performed via the ascending aorta or via axillary artery; axillary artery cannulation is performed by interposition of a Dacron graft and is more often utilized. It allows the extension of the ascending aorta replacement more distally, just proximally to the origin of the brachiocephalic trunk and, moreover, if a brief circulatory arrest is mandatory, antegrade cerebral perfusion is guaranteed by right carotid and vertebral artery perfusion. The right atrium is cannulated for venous drainage. Once the aorta is clamped as distally as possible, the ascending aorta is replaced with a straight Dacron tube graft with a pre-anastomosed bi-tricfurcated vascular prosthesis on its very proximal part. If an open distal main graft body anastomosis is required, it is not critical that an aggressive hemiarch has to be performed, as it will be covered by the stent graft. In case of concomitant surgery, as when an aortic root or valve replacement or coronary bypass is needed, the concomitant surgery is performed as the first part of the procedure. Then the epiaortic vessel debranching is performed individually, starting with the left subclavian artery (Figure 3).

### 2.4. Branched Graft Position

The 2 to 4 limb graft origin should be right above the sinotubular junction to allow a longer landing zone and it is important that the branched graft portion sits antero-laterally, at around 10 o’clock, on the ascending aorta. This orientation allows the displacement of the limbs to the epiaortic vessels anterolaterally (between the aorta and the vena cava) to secure the grafts away from the sternum. To avoid any possible kinking the right pleura can be opened to allow more space for the branched grafts.

### 2.5. Left Subclavian Artery Debranching

The left subclavian artery can sometimes be difficult to reach due to aortic arch aneurysm lateral displacement and it is always prone to dissect or more fragile than the other epiaortic vessels. A preemptive elective carotid-to-subclavian bypass can be a good option in case of an unfavorable anatomy at preoperative CT scan. This procedure can be performed before the hybrid arch repair or immediately after the debranching in case of unexpected unfavorable anatomy. The proximal left subclavian artery is covered with the deployed stent graft in the aortic arch. It can also be proximally ligated during the hybrid arch procedure or coiled endovascularly to prevent a type II endoleak. Another surgical option is to tunnel the first limb to the mid-portion of the subclavian artery and an end-to-side anastomosis is performed through a 4-cm subclavicle incision. In some cases, the left subclavian artery can just be sacrificed without a carotid subclavian bypass, and the stent graft may provide an adequate seal without a type II endoleak. 

### 2.6. Radiopaque Markers

To better visualize the proximal and distal ends of the Dacron tube graft or the reinforcement of the ascending aorta (the landing zone of the future TEVAR) during the endovascular step, we mark each end with radio-opaque thread markers passed around and fixed on to the reinforcement. Another marker is placed around the origin of the branched grafts for a precise deployment of the stent graft immediately above it.

### 2.7. Endovascular Technique

The TEVAR implant is usually done as a second-stage procedure. At the beginning of our study the second endovascular step was delayed until after the full recovery of the patients, from fifteen to thirty days after the surgical stage; since the sudden death of a patient waiting for the second step, the TEVAR is usually implanted one or two days after surgery, when the neurological status and hemodynamic status are stable. In the case of poor peripheral artery access, the stent graft is deployed during the surgical step (single step procedure) in an antegrade fashion through a slave limb of the branched grafts on the ascending aorta. Occasionally, a slave conduit via the common iliac artery is used for an endograft implant. In the case of type I debranching, we always performed a control CT scan after the aorta bending in order to check the new landing zone diameter; TEVAR is usually over-sized by 10% to 20%. The endovascular step was performed under general anesthesia, in the angiographic room, after femoral artery exposure or slave conduit preparation. Ventricular pacing was used to obtain a temporary hypotension for a precise deployment of the endoprosthesis. A pre-curved stiff wire, also commonly used for TAVI, is placed in the left ventricle apex for a better stabilization of the endograft in the ascending aorta during its deployment. The radio-opaque markers positioned at the extremities of the aorta bending and around the origin of the new supra-aortic trunk helped to accurately identify the optimal landing zone, avoiding branching graft occlusion. An occlusion balloon was always used to expand and better adapt the proximal part of the TEVAR to the aortic anatomy. Aortic reinforcement also protects native aorta against rupture or dissection even in case of balloon overinflation. Cerebrospinal fluid drainage was positioned before the endovascular step in the presence of previously treated abdominal aortic aneurysm or non-transposed left subclavian artery. The first cases were treated with the Endofit stent graft (LeMaitre Vascular, Burlington, MA, USA); subsequently we used the Gore TAG stent graft (W. L. Gore & Assoc, Flagstaff, AZ, USA), the Talent stent graft and the Valiant endovascular stent graft (Medtronic Inc., Santa Rosa, CA, USA). Patients underwent serial contrast CT scans before discharge at 3, 6 and 12 months, and annually thereafter. 

## 3. Results

All the patients were affected by aortic arch disease involving zones 0–2 according to the Ishimaru arch map [3]. Nine (24%) patients were symptomatic and 28 (77%) patients underwent surgical and endovascular treatment during the same hospital admission. None of the patients were emergent (no frank rupture or hemodynamic instability). Three (8%) patients required a conduit on common iliac to allow safe retrograde TEVAR introduction. There were no intra-operative deaths and three patients underwent re-exploration for bleeding. One patient, who underwent the type I aortic arch hybrid debranching procedure, died suddenly at home in the waiting period between the supra-aortic debranching and the intended stent-graft placement; no autopsy was done. Thirty day in-hospital rates of adverse neurological events for both the surgical and endovascular procedures were 3% for minor stroke, with no permanent neurological deficit and 0% for permanent paraplegia/paraparesis. In one patient, an axillary vein thrombosis occurred in the perioperative period but after three months of oral anticoagulation, thrombosis was solved. At the beginning of our study, in cases of left subclavian artery involvement in the aneurysm, the left subclavian artery was occluded by a vascular plug immediately after TEVAR implantation to avoid type II endoleak. One patient, with type I aortic arch hybrid debranching, developed an ascending aortic pseudoaneurysm at the site of the debranching. One type I aortic arch hybrid debranching patient, over 80-years-old, developed ascending aortic dissection at follow-up. In one patient, who underwent type II aortic arch hybrid debranching, left carotid artery branch closure was detected at follow-up, without any clinical signs.

**Figure 4:**
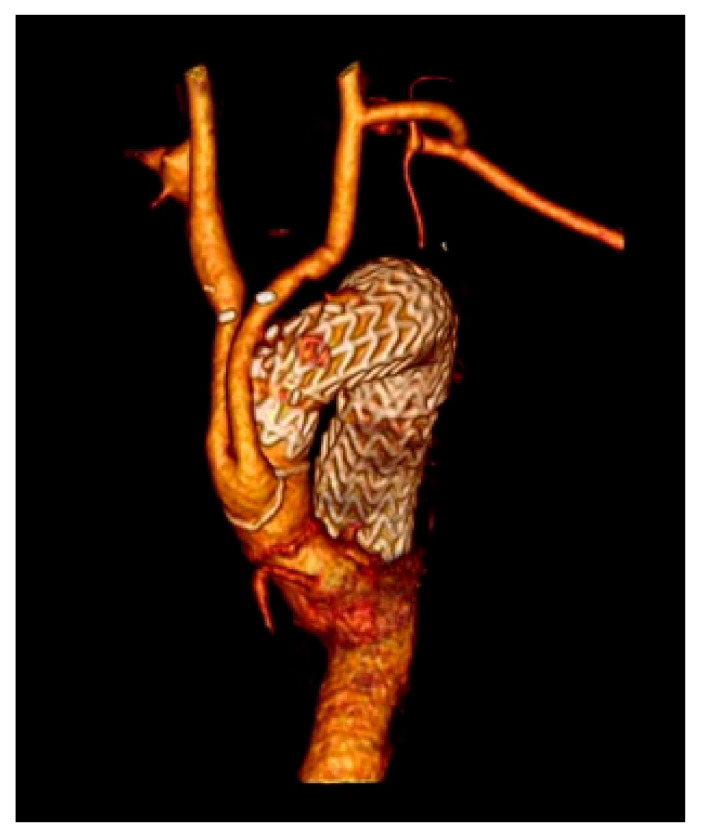
3 years follow-up type II debranching CT scan.

Technically, in 100% of the cases, the endovascular step succeeded. Follow-up was 100% complete with 0% type Ia endoleak rate (Figure 4). 

No additional secondary procedures were necessary in any patient to treat any endoleaks types. In one patient, at 5-year follow-up CT scan, rupture of TEVAR’s stent (Endofit prosthesis) was noted with bulging of the endograft, but without any endoleak; this patient was treated by implanting a new TEVAR (Gore) to cover the bulging part and prevent possible future endoleak (Table 2).

## 4. Discussion

Aortic aneurysms are diagnosed more and more frequently due to better imaging and screening tools. Twelve percent of aortic aneurysm > 6 cm will rupture, without treatment, in one year and fifty percent of these patients will die within five years if they only receive medical treatment [7,8]. 

**Figure 5:**
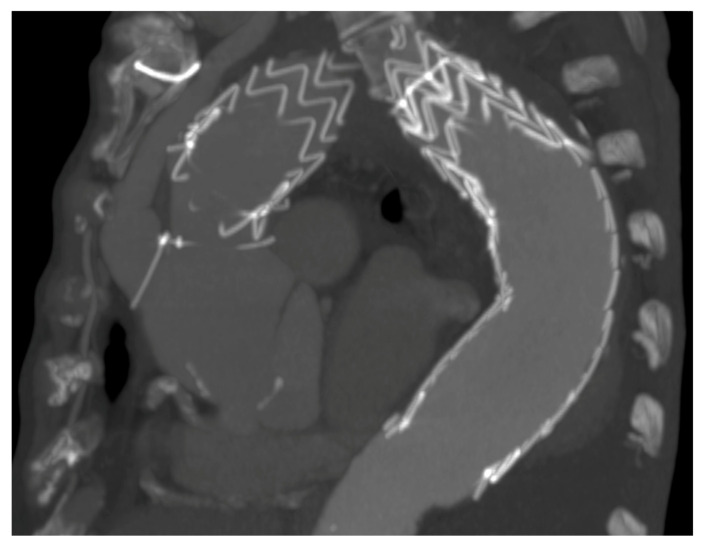
Example of retrograde dissection in a type I debranching.

The gold standard of therapy for patients with extensive aorta arch pathology is still nowadays, surgical [9]. Arch repair was first reported by De Bakey and colleagues [10] in the 1950s, but it was in the 1970s, with the introduction of deep hypothermic circulatory arrest, that acceptable rates of neurological complications and mortality were achieved [11]. A milestone in this context was set by Borst and colleagues in 1983 [12] with the first elephant trunk procedure. The development of selective cerebral perfusion strategies [13], the moderate hypothermic circulatory arrest approach [14], the increasing attention to intraoperative monitoring (transesophageal echocardiography, double invasive arterial, near-infrared spectroscopy-based regional oxygenation, multisite temperature, coagulation and spinal cord perfusion pressure monitoring) [15] and the advent of the frozen elephant trunk technique (conceived by Kato in 1994, introduced in Europe in 2001 and widespread in 2005 with the development of the first commercially available hybrid prosthesis) [16,17,18,19] have led to further improvement in outcomes [20]. Despite all these steps forward, surgery is still related to significant morbidity and mortality rates [21] and not all patients are fit enough to undergo frozen elephant trunk surgery. For this reason, in 1991, Volodos and colleagues performed a hybrid aortic arch repair [22] with the purpose of extending the possibility of treatment to those patients with poor physiological reserve due to comorbidities, taking advantage of both open and endovascular procedures. The core principle behind this treatment relies on endovascular exclusion of the pathology following the creation of an adequate proximal landing zone of at least 25 mm on the inner curve by means of supra-aortic transposition (debranching) of (1) (left subclavian artery), (2) (and left common carotid artery also) or (3) (and innominate artery also) arch vessels [15] to allow stent graft deployment in an increasingly proximal position. Debranching options are multiple and can be performed by means of anatomical or extra-anatomical revascularization, with extra- or intra-thoracic approaches [15] according to the Ishimaru aortic arch zone involved [23]. The main potential advantage of this strategy is the avoidance of aortic cross-clamping, hypothermic cardiac arrest and cardiopulmonary bypass. Different types of debranching procedures have been proposed for zone 0 [24]. Type I allow the avoidance of cardiopulmonary bypass but it is not always feasible, as the aortic side-clamping risks aortic rupture or dissection and future ascending aorta dilation could lead to proximal sealing loss. To avoid the latter, it has been proposed to wrap the proximal landing zone with a Dacron graft [5,6]. Alternatively, it is possible to perform a type II debranching. This approach is mandatory in case of ascending aorta dilation (>37 mm). It needs the institution of cardiopulmonary bypass and aortic cross clamping but it can be conducted in mild hypothermia. Patients presenting with distal aortic arch pathology (zone 1 and 2 proximal neck) are usually considered for an endovascular approach with prior left subclavian artery and/or left carotid artery revascularization by means of extrathoracic bypass. The main problem related to the hybrid approach has been the risk of retrograde dissection (Figure 5) and type Ia endoleak. We have not experienced, in our population, acute retrograde dissection, but in one case a chronic dissection of the sinotubular junction was detected at CT scan follow-up. Proximal endoleak was related, mainly, to the stiffness of the first- and second-generation thoracic stent graft that did not allow to conform itself to the anatomy of the aortic arch, particularly in highly angulated (“gothic”) aortic arches with high stress on the aortic convexity and lack of apposition on the inner curvature resulting in a bird-beak configuration. 

Third generation thoracic endoprosthesis, more conformable to aortic anatomy and specifically designed for deployment on curvature, together with specific endovascular techniques such as appropriate C-arm angulation to avoid parallax, use of a stiff wire leaning on the aortic valve and short period of hypotension, reduce proximal endoleak. 

In our study none of the patients treated had a type IA endoleak immediately after TEVAR implantation or at follow-up. Unique anatomic (angulation and supra-aortic vessels origin) and hemodynamic features (high blood flow and considerable motion) of the aortic arch have made this portion of the aorta the Achille’s heel of TEVAR for a long time. In one patient, treated with first generation endoprosthesis, we found, at five year CT scan follow-up, a fracture of part of the stent endoprosthesis without fabric rupture, probably due to the above mentioned reasons. However, improvements in endograft technology, anatomical customization and embolic protection will expand the use of endovascular arch repair, and as we have seen with the transcatheter aortic valve, a similar endovascular revolution may soon come to the aortic arch. From the first clinical use of stent grafts for abdominal aorta aneurysm repair in 1991 [25], many steps forward have been taken and total endovascular treatment of the aortic arch pathology is already reality. The possibilities available range from the use of parallel grafts (first used in the abdominal aorta in 2003 [26] and, two years later in the aortic arch [27]), in either chimney or snorkel techniques to branched endografts (first performed by Inoue and colleagues in 1999 [28]), passing through in-situ fenestration [29] and fenestrated stent graft (first reported in 2008 [30]). The first technique is an off-the-shelf solution, potentially applicable in urgent or bail-out cases but it has been associated with high endoleak rates [31,32] due to the guttering associated with poor conformability between the main stent-graft and any parallel stents; fenestrated and branched grafts are technically more challenging and are limited by manufacturing times and high cost. 

At this point, given the fact that the aortic arch restitutio-ad-integrum is only possible with surgery and a minimally invasive approach is already feasible for high-risk patients, we wonder what could be the role of hybrid surgery. 

Surgery is the ideal solution, but it is onerous in terms of procedural risk. Although intriguing, the totally endovascular approach is not free from complications (primarily of embolic origin or related to possible retrograde dissection) and some doubts still remain, mainly concerning connective tissue disorders, the possible progression of aneurysmal disease at the level of the proximal landing zone with potentially fatal consequences and the long-term follow-up. For these reasons, type II debranching is preferred whenever the patient can afford a short cardiopulmonary bypass for ascending aorta replacement. In this regard, we should deepen the aspect of the greater pulsatility and flow of the aortic arch and its dynamic strain that could have some consequences in terms of stent patency, migration, kinking and fracture. For all these reasons, despite device improvement and technical progression, total endovascular treatment is nowadays, still limited to a small group of patients with prohibitive surgical risk. Obviously, as in all new techniques, this fact does not allow, at the moment, a fair comparison between open and endovascular surgery and large-scale studies are needed to fill the gap in evidence but we must keep in mind that minimally invasive approaches do not necessarily translate to minimal risk outcomes. 

In our experience the hybrid approach with debranching technique has proved to be safe and effective and early results are promising. Aorta reinforcement for type I debranching is a simple, quick and cost-effective trick. Obviously, a careful preoperative planning is mandatory. Left subclavian artery revascularization, metachronous procedures (debranching and TEVAR) maintaining high arterial pressure during and immediately after the surgical step and the cerebrofluid drainage in case of extended endoprosthesis, contribute to reduce the paraplegia/paraparesis risk. In our series, we have not experienced any paraplegia/paraparesis mainly because all the previous technical aspects were adopted in most of our procedures and only in case of antegrade TEVAR implant, the endovascular step was done simultaneously to the surgical one. 

Of course, stroke remains a major concern in any kind of open, hybrid or endovascular aortic arch treatment strategy and it can be seen as the major important challenge to address in the years to come. Despite careful attention during surgical and endovascular steps we experienced, in our group of patients, one minor stroke in the thirty-day postoperative period. Open, hybrid and endovascular approaches constitute, together, a spectrum of possibilities from the more anatomical to the less invasive, a continuum into which each patient should be inserted in the right place in order to find the solution that allows maximum benefits with minimum risk. This is possible only with a careful and customized evaluation, case by case, and with a deep knowledge of the different techniques, materials and innovations. Modern cardiac surgeons have to acquire and master interventional and vascular skills so that they can control the whole range of possibilities in the grey zone of the aortic arch pathology and offer the patient the best solution. 

At the moment, it is science fiction but who knows if in the future, with the improvement of technology and techniques, the surgical risk will be reduced to the point of considering type II debranching a routine option for young patients with ascending aorta aneurysm and initial aortic arch dilation.

## 5. Conclusions

Hybrid surgery is still a valuable option for aortic arch aneurysm treatment in patients with high surgical risk. It allows the exclusion of the diseased aortic wall by means of endoprosthesis after the creation of a safe proximal landing zone, thus avoiding both the risk of cardio-circulatory arrest and the uncertainties related to the total endovascular approach. Further large-scale studies are needed to better define the application fields of each technique.

## Figures and Tables

**Table 1:** Patient demographics and clinical risk factors (polyvascular disease: one or more claudication, carotid occlusion or >50% stenosis, previous or planned intervention on the abdominal aorta, limb arteries or carotids; COPD: chronic obstructive pulmonary disease).

Demographic	*N*	%
Age, median years	72 (56–78)	
Male gender	29	76.3
Polyvascular disease	21	55.3
Chronic renal failure	13	34.2
COPD	12	31.6
Previous myocardial infarction	10	26.3
Previous cerebrovascular accident	5	13.2
Euroscore, mean ± SD		11.5 ± 5.1
Aortic aneurysm diameter, mean ± SD	5.70 ± 0.80 cm	
Aortic arch pathology Ishimaru zone 0	8	21.1
Aortic arch pathology Ishimaru zone 1	18	47.4
Aortic arch pathology Ishimaru zone 2	12	31.5

**Table 2:** Postoperative complications.

Complications	*N*	%
Early		
Intra-operative deaths, *n* (%)	0	0%
Re-exploration for bleeding	3	8
Minor stroke	1	3
Paraplegia/paraparesis	0	0
Endoleaks	0	0
Axillary vein thrombosis	1	3
Late		
Inter-procedural deaths	1	3
Endoleaks	0	0
Ascending aorta pseudoaneurysm	1	3
Ascending aorta dissection	1	3
Left carotid artery closure	1	3
TEVAR flair and bulging (treated)	1	3

## Data Availability

Data is contained within the article.
